# Eletrochemical immunosensor development for detection of dengue

**DOI:** 10.1186/1753-6561-8-S4-P73

**Published:** 2014-10-01

**Authors:** Isis Prado, , Salvatore De-Simone, Eduardo Ponzio

**Affiliations:** 1 CDTS-FIOCRUZ/INCT-IDN/Laboratório de Bioquímica de Proteínas e Peptídeos, IOC - FIOCRUZ, Av. Brasil 4365, Rio de Janeiro-RJ, Brazil; 2 Universidade Federal Fluminense - Campus Valonguinho, IQ, Outeiro de São João Batista s/n, Niterói-RJ, Brazil

## Background

The development of biosensors for the diagnosis and monitoring of diseases, drug discovery, proteomics, and the environmental detection of pollutants and/or biological agents is an extremely significant problem. Fundamentally, a biosensor is derived from the coupling of a ligand-receptor binding reaction to a signal transducer. In this study we used as a model a peptide marker of DENV (dengue virus) type 1. The electrochemical technique of cyclic voltammetry was performed to detect the signal generated by the interaction between the peptide and antibody from patients' blood samples. Graphite and gold electrodes modified with chitosan film were employed to evaluate the interaction antigen-antibodies. The construction of this immunosensor, able to identify in real time circulating antibodies or antigens can be applied in the diagnosis of dengue and other infectious and parasitic diseases.

## Methods

The synthetic peptide LBPP-D1A (modified or not the N and C-terminal) corresponding to a specific epitope of DENV1 was obtained by F-moc strategy synthesis. This epitope was identified by Spot-synthesis assay using sera of patients. To develop our immunosensor printed electrodes of gold, silver and carbon were used as supports to adsorbed antigenic peptides and capture antibodies from human sera. However as this interaction is electrochemically inert, different chemical conditions and concentrations of peptides were evaluated to obtain a measurable signal. The technique involved the immobilization of the peptide with chitosan film and detection of the signal by cyclic voltammetry under condition of potential -1.0 to 1.0 V, speed of 0.1 V / s and using 200 scans cycles. Other studies involved the modification of the graphite electrode with a film of chitosan prepared in solution of glutaraldehyde and insertion of gold nanoparticles for signal optimization [1].

## Results and conclusions

The best condition of immobilization/detection of the peptide was basic pH and concentrations of 1.0 mg/mL. PBS buffer, pH 7.4, was used as the electrolyte and later added to this serum PBS. Studies with graphite electrode and film of chitosan prepared with glutaraldehyde although useful have shown the need for protection of the carboxylic' hydroxyl group. Studies are in progress, with satisfactory results, using cross-linking epichlorohydrin. Studies on gold electrode were also performed, but with unsatisfactory results, although gold is considered a noble metal, this situation was not a good choice, unlike the graphite electrode, a lamellar structure facilitates the reticular formation of the network more suitable immobilization. Significant results were obtained, however to guarantee the stability and storage of the dispositive sensor, other additional immobilization techniques condition need be evaluated. This study shown that the construction of portable electrochemical immunosensors using specific peptides may be a sensitive, specific and alternative technique for the diagnosis of several infectious and parasitic diseases.
